# Genome characterization of *Trichophyton mentagrophytes* genotype VII strain PG12DES from Italy

**DOI:** 10.1093/mmy/myaf054

**Published:** 2025-06-23

**Authors:** Luca Rossi, Annarita Sorrentino, Prof Caterina Signoretto, Paolo Gaibani

**Affiliations:** Department of Diagnostic and Public Health, Microbiology Section, Verona University, Verona, Italy; Microbiology and Virology Unit, Azienda Ospedaliera Universitaria Integrata Di Verona, Verona, Italy; Department of Diagnostic and Public Health, Microbiology Section, Verona University, Verona, Italy; Microbiology and Virology Unit, Azienda Ospedaliera Universitaria Integrata Di Verona, Verona, Italy; Department of Diagnostic and Public Health, Microbiology Section, Verona University, Verona, Italy; Microbiology and Virology Unit, Azienda Ospedaliera Universitaria Integrata Di Verona, Verona, Italy

**Keywords:** dermatophytes, *Trichophyton mentagrophytes*, genotype VII, terbinafine-resistance, *SQLE* gene, whole-genome sequencing (WGS)

## Abstract

We characterized the genome of the clinical *Trichophyton mentagrophytes* genotype VII strain PG12DES. PG12DES genome harbored different virulence factors, including MEP-1, MEP-2, MEP-3, MEP-4, SUB-6, and ZAF-A. Phylogenetic analysis revealed that PG12DES was clonally related to a *T. mentagrophytes* strain isolated in Moldova in 2017. This study provides the genome characterization of a clinical strain from Italy and broadens the knowledge of the emergent genotype VII.

Dermatophytes are keratinophilic fungi responsible for superficial skin, hair, and nail infections. *Trichophyton mentagrophytes/interdigitale* species complex is one of the major clinically important fungal groups due to its broad host range, widespread distribution, and increasing involvement in antifungal-resistant infections.^1,2^ Since 2002, several cases of *T. mentagrophytes* skin infections have been reported among sex workers, and recently *T. mentagrophytes* genotype VII (TMVII) has been reported as an emerging dermatophyte that causes ringworm, potentially transmitted through sexual contact.^3^ Since 2021, TMVII infections have been reported among men who have sex with men in France and earlier cases were noted among men traveling to Southeast Asia for sexual tourism.^4^

TMVII infections manifest as pruritic, annular, and scaly lesions on the trunk, groin, genitals, and/or face, and terbinafine has been considered the first-line treatment for TMVII infections, as it has shown favorable clinical outcome.^3^ So far, terbinafine-resistant TMVII strains have not been reported.

In this study, we characterized the genome of *T. mentagrophytes* genotype VII strain PG12DES, isolated in Italy. This strain was in 2022 isolated from a clinical sample of skin scale located around the eyes of a young child.

Antifungal susceptibility testing (AFST) was performed using the Sensititre YeastOne YO10 AFST Plate (Thermo Scientific, Cleveland, OH, USA), and MIC for terbinafine was evaluated by the agar-based method according to the CLSI M38 method.^5^

Internal transcribed spacer (ITS) rDNA and *SQLE* sequences were analyzed by Sanger sequencing analysis.^6^ Multiple sequence alignment of ITS and *SQLE* gene was performed with ClustalOmega v1.2.4 and visualized with Unipro UGENE v49.1. Phylogenetic analysis was performed with MEGA v11.0.11.^7^

Whole-genome sequencing of strain PG12DES was performed as previously described.^8^ Briefly, genomic DNA extraction was performed using the Blood and Tissue Kit (Qiagen, Hilden, Germany) from a culture that was grown onto MEA medium. DNA sequencing was conducted using the Illumina iSeq 100 platform (Illumina, San Diego, CA, USA), and paired-end reads were quality checked with FastQC v0.12.1. *De novo g*enome assembly was performed using SPAdes v3.15.2, and assembly metrics were analyzed by QUAST.^9^ The assembly was annotated by using Augustus v.3.5.0 and Helixer v.0.3.5, and gene prediction was manually investigated by BLAST.^10,11^ Virulence factors were investigated by BLAST analysis using the PHI-base (Pathogen–Host Interaction) database for references.^12^ Genome comparison of PG12DES with closely related strains was investigated by using the average nucleotide identity (ANI) calculator.^13^ Using the *T. mentagrophytes* genomes available via NCBI Genome (Supplementary Table 1), a phylogenetic analysis was performed using ParSNP software, as previously described.^14^

AFST showed that the PG12DES strain was susceptible to the antifungal drugs anidulafungin (0.03 mg/l), micafungin (0.015 mg/l), caspofungin (0.06 mg/l), flucytosine (64 mg/l), posaconazole (0.03 mg/l), voriconazole (0.015 mg/l), itraconazole (0.03 mg/l), fluconazole (1 mg/l), amphotericin B (1 mg/l), and terbinafine (<0.2 mg/l).

The phylogenetic analysis is based on ITS sequences derived from 79 *T. mentagrophytes* strains that belong to the different genotypes, and the here-described clinical strain is shown in Supplementary Figure 1. Phylogenetic analysis showed that strain PG12DES clustered closely to *T. mentagrophytes* genotype VII strains.

To identify polymorphisms related to the observed emerging terbinafine resistance among *Trichophyton* strains, the *SQLE* gene was analyzed using the reference allele (NCBI GenBank KU242352). Analysis of the *SQLE* gene showed that strain PG12DES harboured a wild-type *SQLE* gene, therewith confirming the phenotypic AFST results.

Illumina sequencing produced a total of 15 244 292 reads with a length of 300 bp each. Genome assembly generated 306 contigs ranging from 1 043 814 to 1037 bp in length, and a combined total length of 23 117 013 bp. The genome had a GC-content of 47.71%, an N50 of 160 843 bp, and a mean coverage of 40X. Although fragmented, the genomic rearrangement analysis showed that PG12DES was not affected in comparison to the available genomes. Also, annotation analysis showed that a total of 7187 protein-coding DNA sequences (CDSs) were predicted with Augustus in the genome of PG12DES, while this was 8267 predicted CDSs by Helixer. A similar number of CDSs was observed among the annotated *T. mentagrophytes* genomes that have been released so far in the NCBI Genome database.

Previous studies demonstrated that different virulence factors play a key role in the pathogenesis and progression of dermatophyte infections.^15,16^ In this context, analysis of the virulence factors showed that strain PG12DES harboured various virulence factors, including MEP-1, MEP-2, MEP-3, MEP-4, SUB-6, and ZAF-A, while MEP-5 was absent or exhibited truncated proteins (Supplementary Fig. 2)

To investigate the clonal relatedness of PG12DES with the globally collected *T. mentagrophytes* genomes, a phylogenetic analysis based on core genome SNPs was performed. Phylogenetic analysis showed that PG12DES was closely related to strain D15P156 (NCBI GenBank GCA_003664385.1), a *T. mentagrophytes* strain collected in Moldova in 2017 from a patient suffering from tinea capitis having contact with diseased rabbit (Fig. 1).^17^ Detailed SNP analysis of the core genomes showed that PG12DES differed by 4141, 82 073, 85 605, and 86 238 SNPs in comparison to D15P156, TIMM2789, D15P152, and D15P127, respectively. Also, genome comparison with the D15P156 strain showed that PG12DES exhibited an OrthoANIu value of 99.83%, with an average aligned length of 17 009 500 bp.

**Figure 1.**
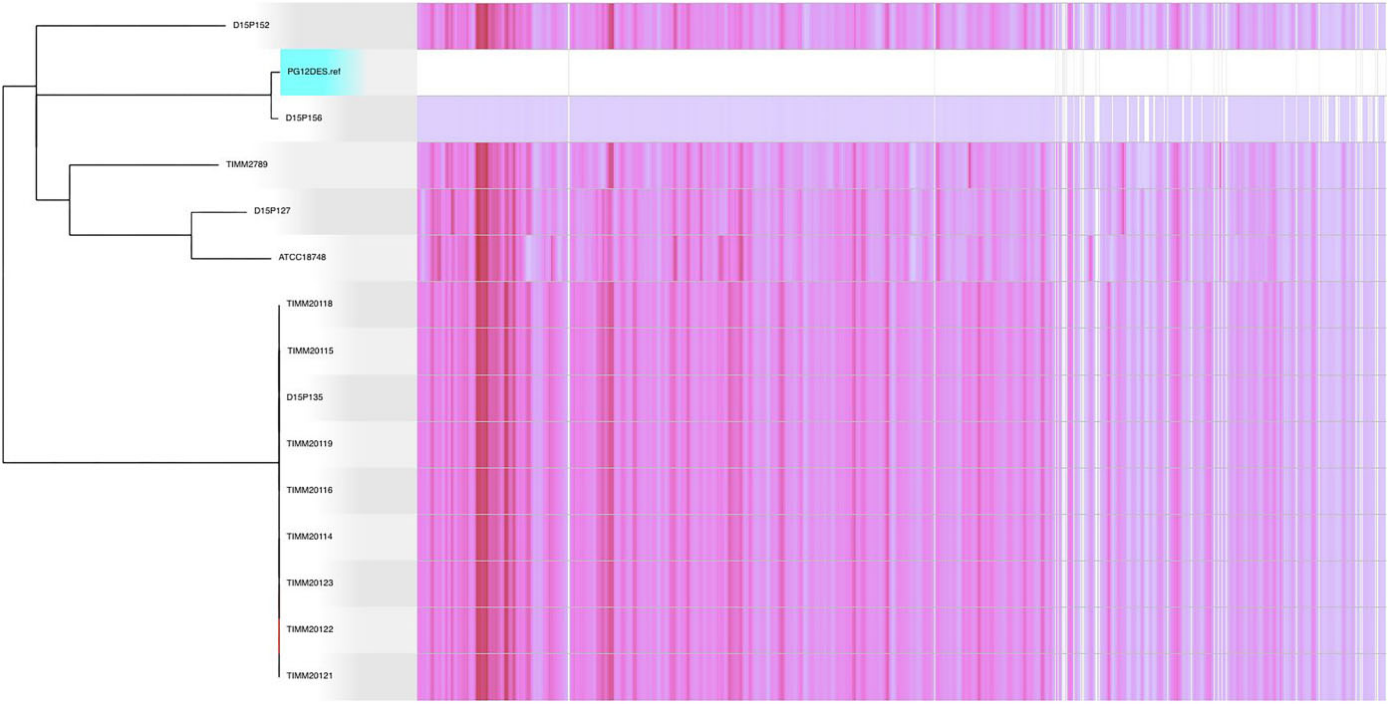
Phylogenetic analysis based on the core genome SNPs of the *T. mentagrophytes* genomes. SNP density plot among *T. mentagrophytes* genomes is shown in the right part of the figure. The PG12DES strain object of this study is highlighted in blue. The scale bar represents substitutions per site.

In the past years, the emergence of *T. mentagrophytes* genotype VII (TMVII) has been reported from France, Germany, and the USA, suggesting a worldwide spread of this emergent genotype.^18–20^ Previous studies demonstrated that TMVII is primarily transmitted from human to human through sexual contact, especially in men who have sex with men.^20,21^

Here, we provide an in-depth genomic analysis of a TMVII strain from Italy. Some limitations should also be acknowledged, including the relatively high number of contigs obtained for the PG12DES genome. In particular, the fragmented genome of PG12DES was due to the intrinsic limitation of the short sequence-read Illumina platform that does not allow to assemble a chromosome-level genome assembly of PG12DES. In this context, the major limitation of this sequencing approach is that we could not rule out the presence of genomic rearrangements between closely related strains, nor could we completely characterize the genomic architecture of this strain.

In conclusion, our results reinforced the worldwide spread of this emergent genotype, expanding the knowledge about the epidemiology of TMVII. In this context, our study highlights the importance of genomic characterization in order to monitor the spread of TMVII, to provide a complete view of the *T. mentagrophytes* epidemiology, and to rapidly identify novel traits of resistance.

## Supplementary Material

myaf054_Supplemental_File

## Data Availability

The genome data of strain PG12DES has been deposited in the NCBI database under BioProject accession number: PRJNA1196900, NCBI BioSample accession number SAMN45682025 and Genome assembly accession number: JBKIZU000000000.
