# Supplementary material for: Genome characterization of Trichophyton mentagrophytes genotype VII strain PG12DES from Italy
Source: Med Mycol. 2025 Jun 23;63(6):myaf054. doi: 10.1093/mmy/myaf054 (PMC12203899; doi:10.1093/mmy/myaf054)
Supplement: myaf054_Supplemental_File [file myaf054_supplemental_file.docx]

**Figure S1.** Phylogenetic tree constructed by Neighbor-Joining method on ITS sequences derived from *T. mentagrophytes* / *T. interdigitale* strains collected in different countries. The evolutionary distance were computed using Maximum Composite Likehood method with unit corresponding to the number of base substitutions per site. In brackets the genotypes for each strain.


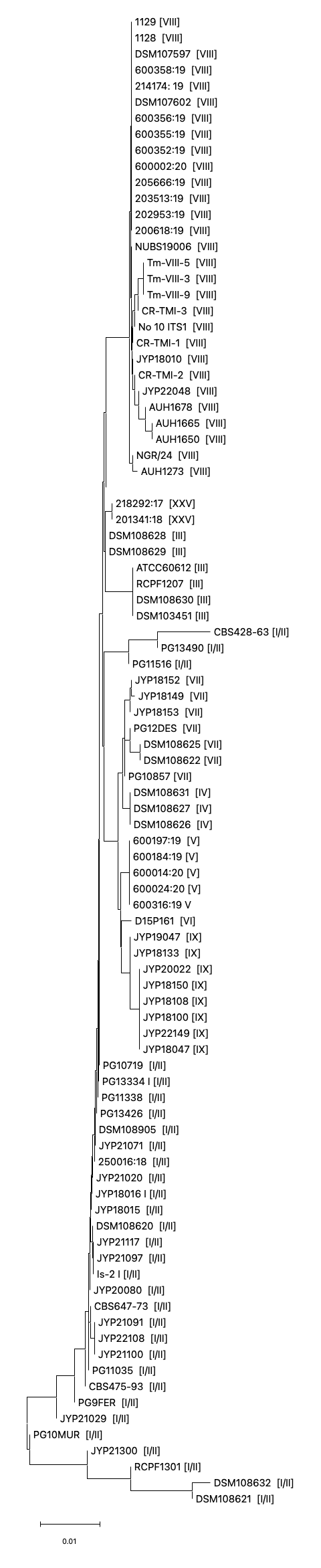


**Figure S2.** Aminoacid alignment of virulence factors in the genome of PG12DES strain in comparison to reference alleles.

**MEP-1**


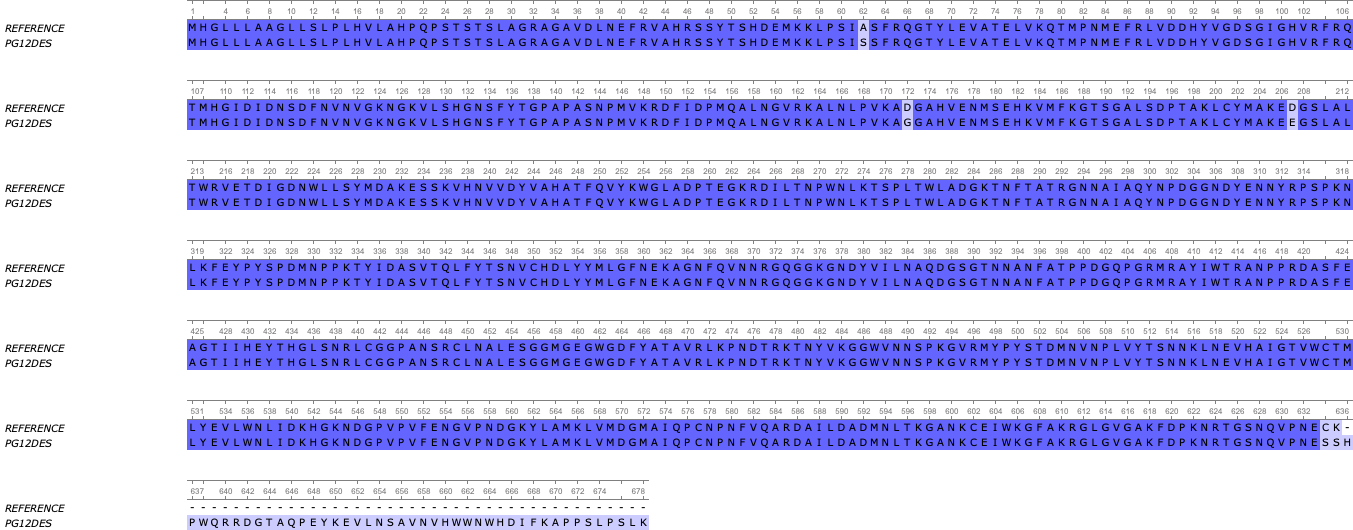


**MEP-2**


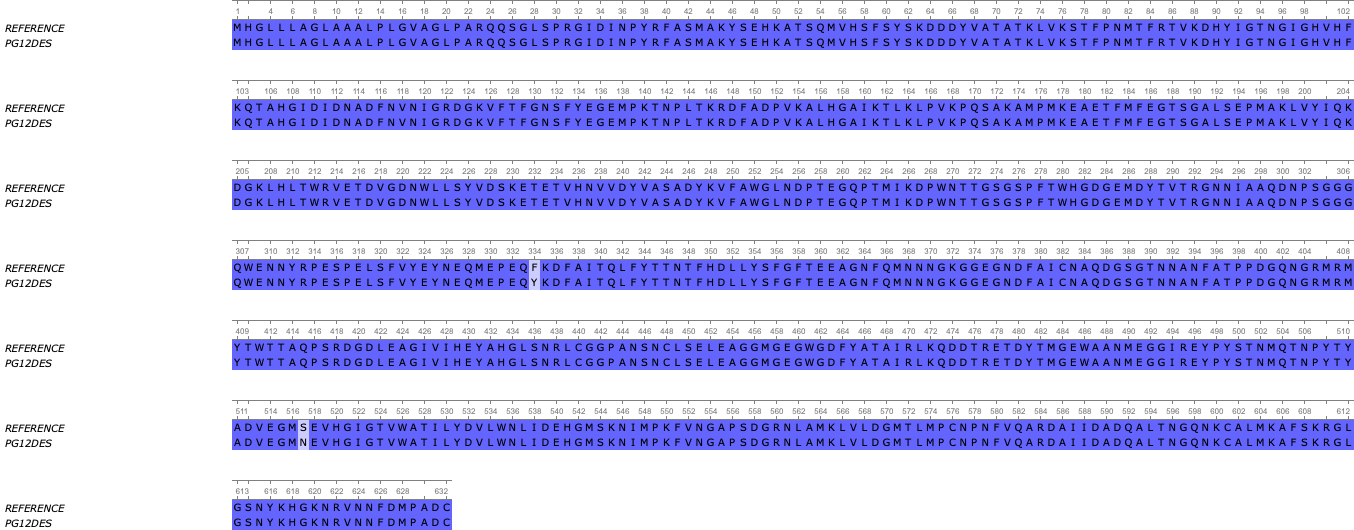


**MEP-3**


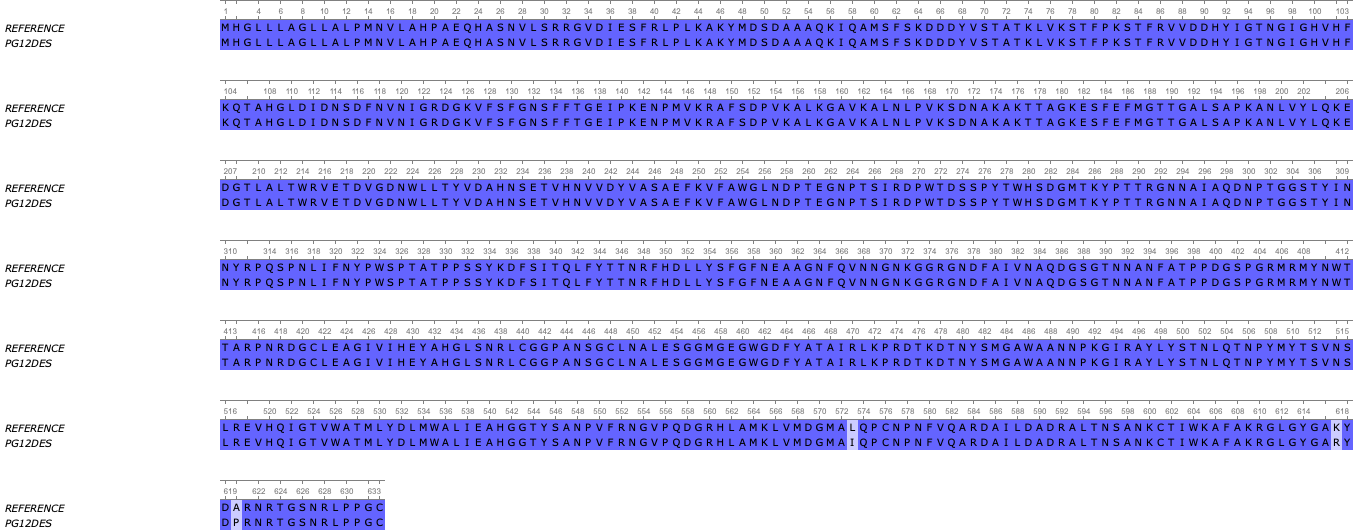


**MEP-4**


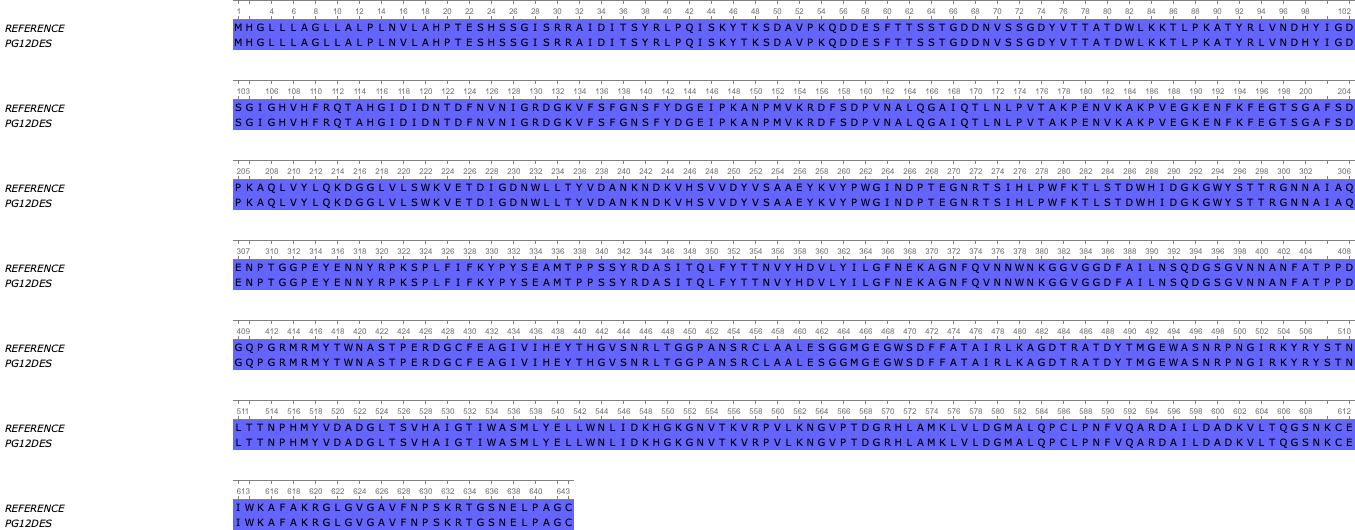


**SUB-6**


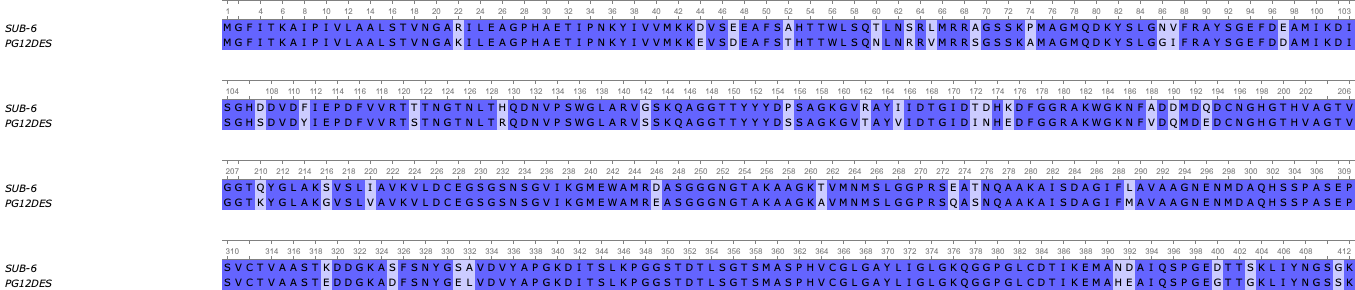


**ZAFA**


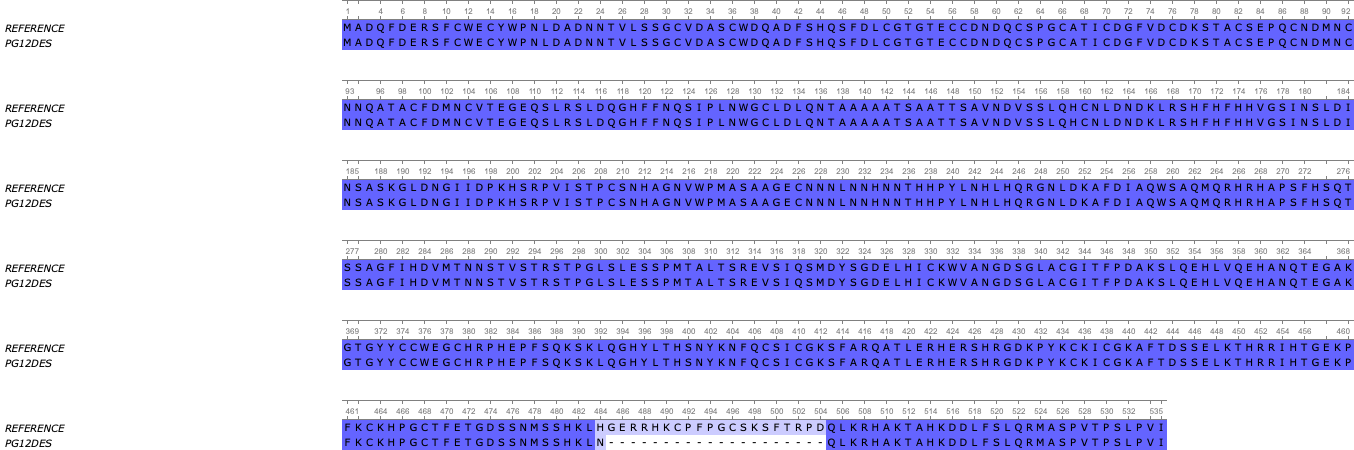


**Table S1.** List of the T. mentagrophytes genomes used for the phylogenetic analysis

| **Strain** | **Acc. no.** | **Country** | **Total Lenght (bp)** | **No. of Contigs** | **N50 (bp)** | **L50** | **G+C (%)** |
| --- | --- | --- | --- | --- | --- | --- | --- |
| ATCC18748 | GCA_045862495.1 | Reference | 23.227.773 | 1.317 | 51.016 | 140 | 47.88 |
| D15P127 | GCA_003664465.1 | Russia | 23.451.472 | 1.212 | 73.243 | 85 | 47.88 |
| D15P135 | GCA_003664455.1 | India | 22.297.183 | 862 | 50.156 | 135 | 48.64 |
| D15P152 | GCA_003664425.1 | Russia | 22.677.904 | 6.731 | 5.157 | 1319 | 48.08 |
| D15P156 | GCA_003664385.1 | Moldova | 23.058.521 | 955 | 68.271 | 98 | 47.98 |
| TIMM2789 | GCA_003118255.1 | Japan | 22.795.542 | 758 | 85.765 | 76 | 48.67 |
| TIMM20114 | GCA_023065905.1 | India | 22.261.634 | 3 | 8.011.651 | 2 | 48.73 |
| TIMM20115 | GCA_023065845.1 | India | 22.303.869 | 8 | 3.672.843 | 3 | 48.69 |
| TIMM20116 | GCA_023065885.1 | India | 22.383.831 | 8 | 4.066.087 | 2 | 48.69 |
| TIMM20118 | GCA_023065865.1 | India | 22.331.438 | 6 | 4.361.119 | 2 | 48.7 |
| TIMM20119 | GCA_023065815.1 | India | 22.297.103 | 8 | 3.646.268 | 3 | 48.7 |
| TIMM20121 | JAUJAB010000000 | India | 22.589.441 | 25 | 2.291.604 | 4 | 48.67 |
| TIMM20122 | GCA_032157395.1 | India | 22.462.006 | 6 | 4.062.023 | 2 | 48.68 |
| TIMM20123 | GCA_032157405.1 | India | 22.613.440 | 23 | 2.205.657 | 4 | 48.67 |
